# Outcome of closed ipsilateral metacarpal fractures treated with mini fragment plates and screws: a prospective study

**DOI:** 10.1007/s10195-011-0166-7

**Published:** 2011-11-12

**Authors:** Ashwani Soni, Anmol Gulati, J. L. Bassi, Daljit Singh, Uttam Chand Saini

**Affiliations:** 1 Department of Orthopedics, Post Graduate Institute of Medical Education and Research, Chandigarh, 160012 India; 2 Department of Orthopedics, Fortis Hospital, Mohali, 160055 India; 3 Department of Orthopedics, Dayanand Medical College and Hospital, Ludhiana, 141001 India

**Keywords:** Metacarpal fracture, Mini fragment plate, Internal fixation

## Abstract

**Background:**

Closed multiple metacarpal fractures are considered highly unstable and are more prone to poor functional outcome. The authors assess the functional outcome of mini fragment plate fixation in closed ipsilateral multiple metacarpal fractures.

**Patients and methods:**

In 21 patients with closed ipsilateral multiple metacarpal fractures treated with open reduction and internal fixation using mini fragment plate, functional outcome was assessed using the American Society for Surgery of the Hand (ASSH) Total Active Flexion (TAF) score and the Disabilities of the Arm, Shoulder, and Hand (DASH) scoring system.

**Results:**

Union rate of 100% was achieved. Functional outcome was excellent in 85.71% (18 of 21) and good in 9% (2 of 21) of patients. Average DASH score was 8.47 (range 1–26). Five cases of infection (two deep, three superficial) were reported, which subsided with dressings and antibiotics.

**Conclusions:**

Plate fixation is a good option for treating closed ipsilateral multiple metacarpal fractures, providing rigid fixation for early mobilization and good functional outcome.

## Introduction

Hand is one of the most frequently injured parts of the body [1]. Functional outcome in case of fractures of small bones of hand depends upon injury severity and management [2]. Ultimate functional outcome is more important than just fracture healing in case of hand fracture [3]. Most hand fractures can be treated by nonoperative methods with good outcome [4, 5]. In the small percentage of unstable hand fractures, results of closed treatment remain unsatisfactory. Closed multiple metacarpal fractures are considered highly unstable and are more prone to poor functional outcome compared with open single metacarpal fracture [6–10].

A small number of prospective studies have been published on treatment of unstable metacarpal and phalangeal fractures using miniature plate (mini plate) and screws [11, 13, 14]. After thorough literature review we did not find any prospective studies in which ipsilateral multiple metacarpal fractures were treated with plating system. We carried out a study in which 21 patients with closed ipsilateral multiple metacarpal fractures were treated with mini fragment plates and screws.

## Patients and methods

A prospective study was conducted from January 2005 to December 2008. Thirty-one consecutive patients with closed ipsilateral multiple metacarpal fractures who were admitted to our institution were enrolled in the study. Patients with two or more metacarpal fractures were included. Two patients died due to associated head injury. Eight patients were lost to follow-up. Finally, a total of 21 patients with 55 metacarpal fractures were included in the study.

The minimum age of the patients in our series was 16 years, and the maximum was 75 years, with mean age of 49.5 years. Of all 21 cases, the majority (>50%) were in either the second or fifth decade of life, with the maximum number of patients in the 21–30-year-old age group, accounting for 28% of total patients. Nineteen patients were male, and two patients were female. Right hand was involved in 11 patients and left in 10 patients. Roadside accidents with high-energy trauma were the mode of injury in most cases (11 cases). The second most common cause of these fractures was assault (seven cases), while few patients suffered these fractures during industrial accidents (two cases) or fall (one case).

Eleven patients had two metacarpal fractures. The most common pattern was ring finger with little finger (five patients), and the least common was little finger with index finger (one patient). Seven patients had three metacarpal fractures, and three patients had four metacarpal fractures.

There are different sizes of plate available to fix metacarpal fractures (1.5-mm screws and titanium mini plates, 2.0-mm screws and stainless-steel AO mini plates, and 2.7-mm screws and stainless-steel AO mini plates). Ultralow-profile plates are also available. We used 2.0-mm stainless-steel AO mini plates with 2.0-mm screws. Souer et al. describe the use of “escape” screws, i.e., a 2.4-mm screw through a 2.0-mm plate, in metaphyseal bone if satisfactory purchase is not obtained with a 2.0-mm screw [9]. However, in our cases we were able to get satisfactory purchase with 2.0-mm screws.

The DASH score and the American Society for Surgery of the Hand (ASSH) Total Active Flexion (TAF) score (Table 1) were used to grade results. The ASSH TAF score grades results as excellent (flexion ≥220), good (flexion 120–80), or poor (flexion ≤80).

**Table 1:** American Society for Surgery of the Hand (ASSH) Total Active Flexion (TAF) score system

Degree of flexion	Rating
TAF from MCPJ to DIPJ: digit 2–5	
>220	Excellent
120–80	Good
<80	Poor
TAF from MCPJ to IPJ: thumb	
>220	Excellent
120–80	Good
<80	Poor

*Clinical Assessment Committee*. Total Active Flexion (TAF) scale, American Society for Surgery of the Hand (ASSH) report. New Orleans, 1976. TAF, total active flexion; MCPJ, metacarpophalangeal joint; DIP, distal interphalangeal joint; IPJ, interphalangeal joint

### Surgical technique

The metacarpal fractures were exposed by dorsal incisions in the space between the involved metacarpals. Extensor tendons were retracted. Fractures were fixed with the plate best suited to the fracture configuration. Reduction and screw sizes were confirmed by image intensifier. Adequate soft tissue closure was achieved over the plate to avoid extensor tendon irritation. Wound was closed without drainage. The hand was rested in elevation for 24–48 h to control pain and swelling, and mobilized actively thereafter. Fracture union was monitored by serial radiographs during fortnightly follow-up visits. Clinical progress in terms of range of movement and complications was recorded at each outpatient visit until healing of fractures, and union was noted. The final range of motion of operated finger was noted in degrees after fracture union. Average follow-up was 1 year.

The study was performed in accordance with the ethical standards of the 1964 Declaration of Helsinki and was approved by the local ethical committee. Written informed consent was obtained from all patients.

## Results

**Fig. 1:**
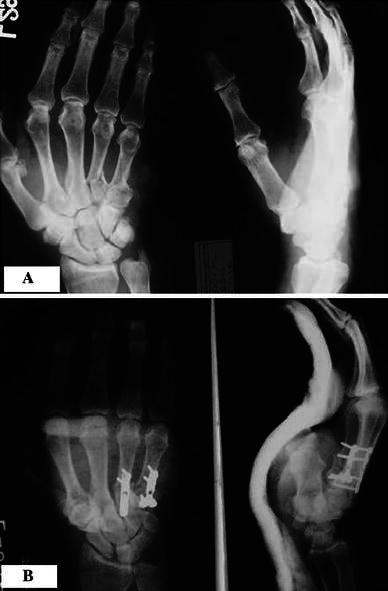
Case 1: **a** preoperative and **b** postoperative X-rays

**Fig. 2:**
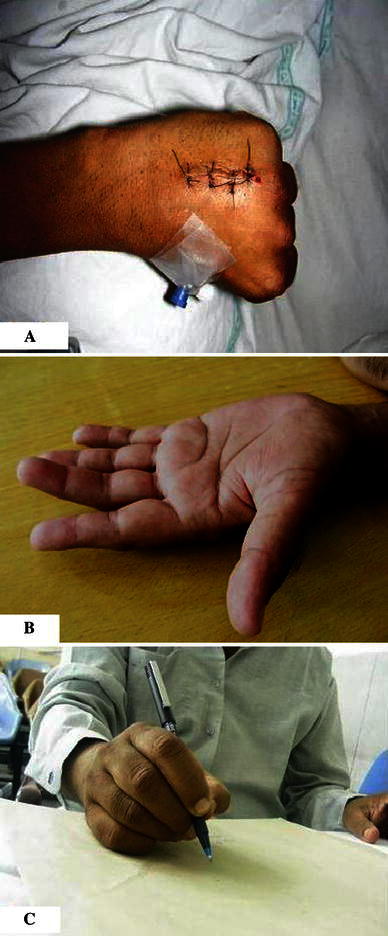
Case 1: **a** full flexion, **b** full extension, and **c** pen-holding, showing pinch

Bone union was seen in all patients, with average period of 6.22 weeks (range 4.5–7.5 weeks). Final functional outcome (as assessed by ASSH TAF score) was excellent in 18 patients, good in 2 patients, and poor in 1 patient. Mean DASH score was 8.47 (range 1–26). The results were satisfactory, as shown in Figs. 1 and 2.

Deep infection was seen in two patients and was managed with daily dressings and antibiotics. Of these two patients, one had four metacarpal fractures and the other had three metacarpal fractures. Superficial infection was seen in three patients and was managed with daily dressings and antibiotics.

There were no cases of angular or rotational displacement. No cases of implant breakage were noted. None of the patients in our study had tendon irritation. This may because we were extra cautious during soft tissue suturing over plate. In none of the patients was implant removal required.

## Discussion

Most hand fractures can be treated by nonoperative methods with good outcome [4, 5]. In the small percentage of unstable hand fractures, results of closed treatment are usually unsatisfactory. Indications for accurate open reduction and internal fixation in hand fractures are few, probably accounting for less than 5% of all hand fractures [15–17]. James reported loss of function in 77% of fingers with unstable phalangeal fracture treated by closed methods [18].

Open reduction and internal fixation of metacarpal fractures with K-wires produces a less rigid fixation with little rotational stability. Protruding ends of the K-wires cause other problems. Interosseous wiring along with K-wire provides more rigid stabilization; however, this method is useful in transverse diaphyseal fractures only.

Metacarpal fracture fixation with external fixator has been described in literature [19–27]. Return of total range of motion was achieved in up to 100% of metacarpal fractures fixed with external fixator by Shehadi et al. [20]. Tun et al. compared the biomechanical properties, clinical versatility, ease of application, and financial cost of seven mini external fixation systems used to treat unstable metacarpal shaft fractures with segmental bone loss [25]. Those authors discouraged routine use of such fixators because of unacceptable loosening at the pin–cement interface during testing and because of difficulties encountered during construction and application.

Transverse and short oblique metacarpal fractures may be splinted with intramedullary wires [28–37]. Flexible bent intramedullary fascicular wires may be used to support oblique fractures. In a study of 21 metacarpal fractures, a J-shaped nail formed from a curved 2.0-mm-diameter Kirschner wire bent sharply at the proximal end was found to be useful in neck or transverse shaft fractures of the metacarpals without concomitant injuries such as severe soft tissue damage [31]. A recent uncontrolled retrospective consecutive study of 22 metacarpal fractures suggested that transcutaneous intramedullary wire fixation of oblique extra-articular metacarpal shaft fracture wires achieves good results and has few complications [36]. In a study of 52 consecutive closed, displaced, extra-articular metacarpal fractures, results of intramedullary nail (IMN) fixation were compared with those of plate–screw (PS) fixation. No significant differences in clinical outcomes were found, but the incidences of loss of reduction, penetration to the metacarpal–phalangeal joint, and secondary surgery for hardware removal in the operating room were much higher in the IMN group [37].

In the literature, several studies have reported satisfactory results for unstable metacarpal and phalangeal fractures fixed with AO mini plates and screws [11, 12, 27, 38–47]. In literature, we found only one study, by Souer et al., in which results of plate fixation in closed ipsilateral multiple metacarpal fractures were evaluated [9]. The study was retrospective, unlike our study. They found total active motion (TAM) >230° in 18 of 19 patients. They had two patients with plate-related complications and one delayed union. Their functional results as evaluated by TAM were quite similar to our results. Gupta et al., in their prospective study, divided patients with fractured metacarpals into four groups. They found TAM >230 in all patients in the group where fracture was fixed with plates [27]. Dabezies and Schutte reported no complications in 27 unstable metacarpal fractures fixed with plates [40]. Our low complication rate is similar to their results. Other authors have reported that patients with open fractures and severe soft tissue injury have high rate of complications [45, 48–50]. Nonunion and delayed union are infrequent findings in metacarpal fractures. Souer et al. reported 1 of 19 patients having delayed union [9]; the patient was a smoker. Page and Stern [49] found nonunion in 1 of 66 patients, and Stern et al. [45] found nonunion in 3 of 17 patients. Their low rate of nonunion and delayed union was similar to our results.

Infection was seen in 5 of 21 patients. Two patients had deep and three had superficial infection. In all three patients who had superficial infection, there was discharge from the wound from postoperative day 1, which was settled within postoperative day 3 with daily dressings and antibiotics. In two cases with deep infection, the discharge persisted up to postoperative day 7. Though the rate of infection was quite high, all patients were managed with dressings and antibiotics, and the final outcome was not affected.

In closed multiple metacarpal fractures, plate fixation is a good option for several reasons. These fractures are highly unstable, and stable fixation is required in these fractures [9]. Metacarpal length is very likely to be shortened in multiple metacarpal fractures, causing instability [6, 7]. This effect is greater in internal metacarpals (third and fourth metacarpals) than in border metacarpals (second and fifth metacarpals), because the latter are anchored on both sides of the metacarpal head [8]. Closed ipsilateral multiple metacarpal fractures are frequently associated with more soft tissue injury as compared with single fracture, making them more susceptible to stiffness and poor functional results. Osteosynthesis using miniature plates and screws in these unstable fractures produces anatomical reduction of fractures with stabilization that is rigid enough to allow early mobilization of adjacent joints without allowing loss of reduction, thereby preventing stiffness and hence good functional results.

In our study, we found a 100% union rate, with 85.71% (18 of 21) excellent and 9% (2 of 21) good results according to the American Society for Surgery of the Hand (ASSH) Total Active Flexion (TAF) score. Mean DASH score was 8.47 (range 1–26). Rigid and stable fixation with mini plates allowed early mobilization, which prevented stiffness and achieved good functional result. Though the infection rate was high, it was managed with dressings and antibiotics in all patients.

In conclusion, plate fixation is a good option for treating closed multiple metacarpal fractures, providing rigid fixation for early mobilization and good functional outcome.

## References

[1] Emmett JE, Breck LW (1958). A review analysis of 11, 000 fractures seen in a private practice of orthopaedic surgery, 1937–1956. J Bone Joint Surg Am.

[2] Drenth DJ, Klasen HJ (1998). External fixation for phalangeal and metacarpal fractures. J Bone Joint Surg Br.

[3] Brenwald J (1987). Bone healing in the hand. Clin Orthop Relat Res.

[4] Barton N (1989). Conservative treatment of articular fractures in the hand. J Hand Surg Am.

[5] Wright TA (1968). Early mobilization in fractures of the metacarpals and phalanges. Can J Surg.

[6] Eglseder WA, Juliano PJ, Roure R (1997). Fractures of the fourth metacarpal. J Orthop Trauma.

[7] Meunier M, Hentzen E, Ryan M (2004). Predicted effects of metacarpal shortening on interosseous muscle function. J Hand Surg Am.

[8] Freeland AE, Orbay JL (2006). Extraarticular hand fractures in adult. Clin Orthop Relat Res.

[9] Souer JS, Mudgal CS (2008). Plate fixation in closed ipsilateral multiple metacarpal fractures. J Hand Surg Eur.

[10] Smith RJ (1974). Balance and kinetics of the fingers under normal and pathological conditions. Clin Orthop Relat Res.

[11] Agarwal AK, Pickford MA (2006). Experience with a new ultralow-profile osteosynthesis system for fractures of the metacarpals and phalanges. Ann Plast Surg.

[12] Bosscha K, Snellen JP (1993). Internal fixation of metacarpal and phalangeal fractures with AO minifragment screws and plates: a prospective study. Injury.

[13] Pun WK, Chow SP, So YC (1991). Unstable phalangeal fractures: treatment by A.O. screw and plate fixation. J Hand Surg Am.

[14] Omokawa S, Fujitani R, Dohi Y (2008). Prospective outcomes of comminuted periarticular metacarpal and phalangeal fractures treated using a titanium plate system. J Hand Surg Am.

[15] Amadio PC, Jupiter JB (1991). Fractures of the hand and the wrist. Flynn’s hand surgery.

[16] Stern PJ, Green DP (1999). Fractures of the metacarpals and phalanges. Operative hand surgery.

[17] Barton NJ (1984). Fractures of the hand. J Bone Joint Surg Br.

[18] James JIP (1962). Fractures of the proximal and middle phalanges of the fingers. Acta Orthop Scand.

[19] Parsons SW, Fitzgerald JA, Shearer JR (1992). External fixation of unstable metacarpal and phalangeal fractures. J Hand Surg Br.

[20] Shehadi SI (1991). External fixation of metacarpal and phalangeal fractures. J Hand Surg Am.

[21] Schuind F, Donkerwolcke M, Burny F (1991). External minifixation for treatment of closed fractures of the metacarpal bones. J Orthop Trauma.

[22] Pritsch M, Engel J, Farin I (1981). Manipulation and external fixation of metacarpal fractures. J Bone Joint Surg Am.

[23] Büchler U (1994) The small AO external fixator in hand surgery. Injury 25 (Suppl 4):S-D55-63 10.1016/0020-1383(95)90131-0 7868198

[24] Pennig D, Gausepohl T, Mader K (2000). The use of minimally invasive fixation in fractures of the hand–the minifixator concept. Injury.

[25] Tun S, Sekiya JK, Goldstein SA (2004). A comparative study of mini-external fixation systems used to treat unstable metacarpal fractures. Am J Orthop (Belle Mead NJ).

[26] Margić K (2006). External fixation of closed metacarpal and phalangeal fractures of digits. A prospective study of one hundred consecutive patients. J Hand Surg Br.

[27] Gupta R, Singh R, Siwach R (2007). Evaluation of surgical stabilization of metacarpal and phalangeal fractures of hand. Indian J Orthop.

[28] Gonzalez MH, Igram CM, Hall RF (1995). Flexible intramedullary nailing for metacarpal fractures. J Hand Surg.

[29] Orbay JL, Indriago I, Gonzalez E (2002). Percutaneous fixation of metacarpal fractures. Op Tech Plast Reconstruct Surg.

[30] Gonzalez MH, Hall RF (1996). Intramedullary fixation of metacarpal and proximal phalangeal fractures of the hand. Clin Orthop Relat Res.

[31] Itadera E, Hiwatari R, Moriya H (2008). Closed intramedullary fixation for metacarpal fractures using J-shaped nail. Hand Surg.

[32] Balfour GW (2008). Minimally invasive intramedullary rod fixation of multiple metacarpal shaft fractures. Tech Hand Up Extrem Surg.

[33] Orbay JL, Touhami A (2006). The treatment of unstable metacarpal and phalangeal shaft fractures with flexible nonlocking and locking intramedullary nails. Hand Clin.

[34] Downing ND, Davis TR (2006). Intramedullary fixation of unstable metacarpal fractures. Hand Clin.

[35] Orbay J (2005). Intramedullary nailing of metacarpal shaft fractures. Tech Hand Up Extrem Surg.

[36] Faraj AA, Davis TR (1999). Percutaneous intramedullary fixation of metacarpal shaft fractures. J Hand Surg Br.

[37] Ozer K, Gillani S, Williams A (2008). Comparison of intramedullary nailing versus plate-screw fixation of extra-articular metacarpal fractures. J Hand Surg Am.

[38] Chen SH, Wei FC, Chen HC (1994). Miniature plates and screws in acute complex hand injury. J Trauma.

[39] Ford DJ, el-Hadidi S, Lunn PG (1987). Fractures of the metacarpals: treatment by A. O. screw and plate fixation. J Hand Surg Br.

[40] Dabezies EJ, Schutte JP (1986). Fixation of metacarpal and phalangeal fractures with miniature plates and screws. J Hand Surg Am.

[41] Büchler U, Fischer T (1987). Use of a minicondylar plate for metacarpal and phalangeal periarticular injuries. Clin Orthop Relat Res.

[42] Diwaker HN, Stothard J (1986). The role of internal fixation in closed fractures of the proximal phalanges and metacarpals in adults. J Hand Surg Br.

[43] Hastings H, Carroll C (1988). Treatment of closed articular fractures of the metacarpophalangeal and proximal interphalangeal joints. Hand Clin.

[44] Melone CP (1986). Rigid fixation of phalangeal and metacarpal fractures. Orthop Clin North Am.

[45] Stern PJ, Wieser MJ, Reilly DG (1987). Complications of plate fixation in the hand skeleton. Clin Orthop Relat Res.

[46] Thakore HK (1986). Osteosynthesis for the unstable fracture of the hand. J Hand Surg Br.

[47] Trevisan C, Morganti A, Casiraghi A (2004). Low severity metacarpal and phalangeal fractures treated with miniature plates and screws. Arch Orthop Trauma Surg.

[48] Fusetti C, Meyer H, Borisch N (2002). Complications of plate fixation in metacarpal fractures. J Trauma.

[49] Page SM, Stern PJ (1998). Complications and range of motion following plate fixation of metacarpal and phalangeal fractures. J Hand Surg Am.

[50] Ouellette EA, Freeland AE (1996). Use of the minicondylar plate in metacarpal and phalangeal fractures. Clin Orthop.

